# Feasibility and Optimal Time Point of [^68^Ga]Gallium-labeled Prostate-specific Membrane Antigen Ligand Positron Emission Tomography Imaging in Patients Undergoing Cytoreductive Surgery After Systemic Therapy for Primary Oligometastatic Prostate Cancer: Implications for Patient Selection and Extent of Surgery

**DOI:** 10.1016/j.euros.2022.04.003

**Published:** 2022-05-05

**Authors:** Nicolai Huebner, Sazan Rasul, Pascal Baltzer, Paola Clauser, Karl Hermann Grubmüller, Markus Mitterhauser, Marcus Hacker, Axel Heidenreich, Pawel Rajwa, Harun Fajkovic, Shahrokh F. Shariat, Bernhard Grubmüller

**Affiliations:** a Department of Urology, Medical University of Vienna, Vienna, Austria; b Working Group for Diagnostic Imaging in Urology (ABDU), Austrian Association of Urology (ÖGU), Vienna, Austria; c Department of Biomedical Imaging and Image Guided Therapy, Division of Nuclear Medicine, Medical University of Vienna, Vienna, Austria; d Department of Biomedical Imaging and Image Guided Therapy, Division of General and Pediatric Radiology, Medical University of Vienna, Vienna, Austria; e Department of Urology and Andrology, University Hospital Krems, Karl Landsteiner University of Health Sciences, Krems, Austria; f Ludwig Boltzmann Institute Applied Diagnostics, Vienna, Austria; g Department of urology, Uro-oncology, Robot-Assisted and Specialized Surgery, University of Cologne, Cologne, Germany; h Department of Urology, Medical University of Silesia, Zabrze, Poland; i Karl Landsteiner Institute of Urology and Andrology, Vienna, Austria; j Department of Urology, University of Texas Southwestern, Dallas, TX, USA; k Department of Urology and Division of Medical Oncology, Weill Medical College of Cornell University, New York, NY, USA; l Department of Urology, Second Faculty of Medicine, Charles University, Prague, Czech Republic; m Institute for Urology and Reproductive Health, I.M. Sechenov First Moscow State Medical University, Moscow, Russia

**Keywords:** Prostate-specific membrane antigen, Prostate cancer, Cytoreductive surgery, Radical prostatectomy, Hormone therapy

## Abstract

**Background:**

Prostate-specific membrane antigen (PSMA) targeted molecular imaging using positron emission tomography (PET) has significantly improved the diagnosis and treatment of prostate cancer (PCA).

**Objective:**

To assess the feasibility and compare the diagnostic accuracy of [^68^Ga]Ga-PSMA-11 PET images taken at baseline, before the initiation of systemic treatment and preoperative images, using histopathology after cytoreductive surgery as reference.

**Design, setting, and participants:**

We identified 20 patients in our prospectively maintained database with primary oligometastatic PCA who underwent cytoreductive radical prostatectomy and superextended pelvic lymph node dissection after systemic therapy, who had baseline and preoperative [^68^Ga]Ga-PSMA-11 PET imaging available.

**Outcome measurements and statistical analysis:**

We performed a region-based analysis to determine the diagnostic accuracy of imaging, using pathology as a reference. Regions were predefined as prostate, internal iliac left/right, obturator left/right, external iliac left/right, common iliac left/right, and presacral.

**Results and limitations:**

Sensitivity, specificity, negative predictive value (NPV), positive predictive value (PPV), and diagnostic effectiveness were, respectively, 95.65%, 78.22%, 98.39%, 57.89%, and 83.00% for baseline [^68^Ga]Ga-PSMA-11 PET, compared to 56.52%, 98.05%, 88.30%, 89.66%, and 88.50% for preoperative [^68^Ga]Ga-PSMA-11 PET. On a receiver operating characteristic analysis, the diagnostic accuracy of baseline [^68^Ga]Ga-PSMA-11 PET with an area under the curve (AUC) of 0.87 (95% confidence interval [CI] 0.83–0.92) was significantly better than that of preoperative [^68^Ga]Ga-PSMA-11 PET after systemic therapy with an AUC of 0.77 (95% CI 0.70–0.85, *p* = 0.01).

**Conclusions:**

Baseline imaging, [^68^Ga]Ga-PSMA-11 PET has significantly better diagnostic accuracy, sensitivity, and NPV than images obtained preoperatively, in systemically pretreated patients. If a patient is suitable for local treatment and complete resection of the residual tumor is intended, [^68^Ga]Ga-PSMA-11 PET images taken prior to systemic therapy are significantly more accurate in selecting the relevant lymph nodes for resection.

**Patient summary:**

We found that prostate-specific membrane antigen positron emission tomography (PSMA-PET) imaging used early, before hormonal therapy or chemotherapy, provides more accurate information about the spread of the disease, than if used immediately before surgery but after hormonal therapy or chemotherapy. Early use of PSMA-PET has the potential to improve therapy also at later stages of the disease.

## Take Home Message

Prostate-specific membrane antigen positron emission tomography baseline imaging provides better information on residual disease after systemic therapy than preoperative imaging. Early application should be considered in high-risk localized or oligometastatic patients, as it has the potential to guide local therapy even at later stages.

## 1 Introduction

The introduction of prostate-specific membrane antigen (PSMA) targeted molecular imaging using positron emission tomography (PET) has significantly enriched the diagnostic and therapeutic landscape of prostate cancer (PCA). It is an accurate imaging modality for local, regional, and distant staging in primary, recurrent, metastatic, as well as castration-resistant PCA [1], [2], [3], [4], [5], [6], [7], [8]. The most widely used and only guideline-recommended indication is at the time of biochemical recurrence (BCR) after local treatment with curative intent [4], [9], [10], [11], to differentiate between local and metastatic recurrence.

In patients with presumed high-risk localized disease, the use of PSMA-PET has led to a disease state shift, partly due to its improved sensitivity for oligometastases compared with standard imaging. Many oligometastatic PCA patients diagnosed by PSMA-PET show no evidence of systemic disease when assessed using standard imaging, which includes a bone scan (BS) and computer tomography (CT) [2], [7], [8], [12], [13]. This has led to an increased use of local radical therapy in patients with oligometastatic PCA, commonly after a response to systemic therapy [14], [15].

In a previous prospective study, we have demonstrated that preoperative [^68^Ga]gallium-PSMA^HBED-CC^ conjugate 11-PET ([^68^Ga]Ga-PSMA-11 PET) had high diagnostic accuracy for local staging in systemic therapy-naïve patients [2]. We hypothesized that the added information gained through PSMA-targeted molecular imaging might improve clinical decision-making for patients undergoing cytoreductive surgery after systemic therapy for oligometastatic PCA, potentially helping to fine-tune the tailoring of local therapy. However, as the effects of systemic therapy might reduce the sensitivity of preoperative [^68^Ga]Ga-PSMA-11 PET, the ideal time point of its use remains unclear.

The aim of this study was to assess the feasibility and compare the diagnostic accuracy of [^68^Ga]Ga-PSMA-11 PET images taken at baseline and before the initiation of systemic treatment, and preoperative images, using histopathology after cytoreductive surgery as reference.

## 2 Patients and methods

### 2.1 Patient selection

This was an ethics-approved retrospective analysis of a prospectively maintained database (NCT02971358). We queried our database of patients treated with radical prostatectomy (RP) and superextended pelvic lymph node (LN) dissection (sePLND) for high-risk locally advanced or primary oligometastatic PCA. Oligometastatic PCA was defined as five or fewer metastases in LNs or bone on conventional imaging using CT and BS. Patients, who were treated with androgen deprivation therapy (ADT) alone, in combination with docetaxel, or in combination with enzalutamide prior to surgery, and had baseline as well as preoperative staging performed using [^68^Ga]Ga-PSMA-11 PET–based hybrid imaging, were included in the study. Patients with incomplete data regarding baseline disease characteristics and disease progression during systemic treatment, as well as those who received radiation to the pelvis were excluded. Informed consent was obtained from all participants included in this study. All participants gave their consent for the results to be published.

### 2.2 Intervention and data collection

All patients were diagnosed with primary oligometastatic PCA using [^68^Ga]Ga-PSMA-11 PET–based hybrid imaging (PET/CT or PET/magnetic resonance imaging [MRI]) as baseline imaging. All patients then went on to receive systemic treatment and were still actively receiving ADT with testosterone levels below the castration threshold at the time of surgery. Systemic treatment consisted of ADT, with luteinizing-hormone-releasing-hormone agonists or antagonists alone, in combination with six cycles of docetaxel (75 mg/m^2^ body surface area, every 3 wk) or in combination with enzalutamide.

Preoperative imaging was performed within 1 mo prior to surgery. All patients received [^68^Ga]Ga-PSMA-11 PET/MRI as preoperative imaging on an integrated PET/MRI system (Biograph mMR; Siemens Healthineers), composed of an MRI-compatible PET detector integrated with a 3.0-Tesla whole-body MRI scanner, at our institution. The PET/MRI protocol has been described previously [4]. In brief, a local PET and multiparametric MRI (mpMRI) protocol of the prostate in accordance with international guidelines [16] without an endorectal coil was performed. The local PET/mpMRI was followed by a whole-body scan from the skull base to the knees. A detailed description of the complete PET/MRI protocol is given in the Supplementary material.

All patients then underwent open cytoreductive RP with meticulous sePLND up to the level of the inferior mesenteric artery, including the nodes of the presacral, common iliac, external iliac, internal iliac, and obturator regions. During surgery, every region was sent as a different specimen to allow the correct allocation of nodes. The ten predefined regions were the prostate, obturator right, internal iliac right, external iliac right, common iliac right, obturator left, internal iliac left, external iliac left, common iliac left, and presacral and aortic bifurcation (up to the inferior mesenteric artery). All specimens were reviewed by a dedicated uropathologist at our center.

For this study, all images were re-reviewed by a specialist with expert-level experience at our center using the Hermes hybrid 3D (Hermes Medical Solutions), blinded to the pathologic results, and graded positive or negative for each individual region. For preoperative [^68^Ga]Ga-PSMA-11 PET/MRI, the images were graded in two steps: MRI alone and then using the [^68^Ga]Ga-PSMA-11 PET/MRI images separately and independently. Whole-body radiologic assessment of MRI was performed according to Response Evaluation Criteria in Solid Tumours (RECIST) version 1.1 criteria [17], and a focal uptake above the surrounding background in a morphologically visible structure (eg, LN of any size or bone) or corresponding areas of restricted diffusion capacity in MRI diffusion-weighted imaging were assessed as a positive finding for hybrid assessment. Local mpMRI of the prostate was graded according to the Prostate Imaging Reporting and Data System (PI-RADS) version 2 criteria [16], and any focal PSMA uptake within the prostate was considered positive, regardless of the PI-RADS score.

### 2.3 Statistical analysis

#### 2.3.1 Primary endpoint

The primary endpoint of the study was to compare the diagnostic accuracy of baseline [^68^Ga]Ga-PSMA-11 PET hybrid imaging with preoperative [^68^Ga]Ga-PSMA-11 PET/MRI after systemic therapy, on a region-based analysis. To assess this, contingency tables were used with histopathologic findings as a reference standard. Diagnostic accuracy, sensitivity, specificity, negative and positive predictive values, as well as likelihood ratios (LRs) were calculated. A receiver operating characteristic (ROC) analysis was performed, and the area under the curve (AUC) was calculated for both examinations and compared.

#### 2.3.2 Secondary endpoints

Multiple secondary endpoints were explored. Diagnostic accuracy was assessed for preoperative MRI alone and compared with that of [^68^Ga]Ga-PSMA-11 PET/MRI by the same methods as the primary endpoint. Diagnostic accuracy for baseline and preoperative [^68^Ga]Ga-PSMA-11 was also assessed on a per-patient level, with and without the prostate region. Additionally, we performed an exploratory logistic regression analysis for the association of pathologic complete response with preoperative prostate-specific antigen (PSA), duration of ADT, docetaxel, enzalutamide, and radiographic complete response, as well as for the association of pathologic negative LNs and preoperative PSA, duration of ADT, docetaxel, enzalutamide, and radiographic complete response in LNs.

Owing to the exploratory nature of our study, statistical significance was considered for *p* < 0.05, but not in a confirmatory matter. Thus, no adjustments for multiplicity were performed. All tests were two sided and conducted using STATA-14 (StataCorp, College Station, TX, USA).

## 3 Results

The final analysis contained 20 patients. Baseline characteristics of patients are shown in Table 1. At diagnosis, the median PSA was 37 ng/ml, patients were diagnosed with International Society of Urological Pathology 3, 4, or 5, and had locally advanced tumors. Nineteen patients (95%) had evidence of pelvic LN metastasis at diagnosis and eight (40%) had distant metastases, with three having bone metastases only (M1b), two having extrapelvic LNs (M1a), and three having both (M1b and M1a). None of the patients had visceral metastasis. Patients received ADT for a median duration of 8.5 mo, 11 patients also received six cycles of docetaxel, and six patients received enzalutamide. The median PSA at the time of preoperative [^68^Ga]Ga-PSMA-11 PET was 0.39 ng/ml. During surgery, residual tumor in the prostate was present in 17 (85%) patients, and positive LNs were found in ten (50%) patients. Three patients (15%) achieved a complete pathologic response in the prostate and LNs; one received ADT and docetaxel, and two ADT and enzalutamide prior to surgery. Of the 19 patients who had positive pelvic LNs at baseline staging, 12 had a radiographic complete response on [^68^Ga]Ga-PSMA-11 PET (63.16%) and nine had a pathologic complete response on sePLND (47.37%).

**Table 1:** Clinical and pathologic parameters of 20 patients undergoing cytoreductive radical prostatectomy and extended pelvic lymph node dissection for oligometastatic prostate cancer after systemic therapy

Baseline parameters at diagnosis and initial staging using [^68^Ga]Ga-PSMA-11 PET	
Age (yr), median (IQR)	64.5 (60–68)
PSA (ng/ml), median (IQR)	37 (13.7–91)
ISUP, *n* (%)	
3	1 (5.0)
4	11 (55.0)
5	8 (40.0)
cT, *n* (%)	
2	1 (5.0)
3a	5 (25.0)
3b	10 (50.0)
4	4 (20.0)
cN, *n* (%)	
0	1 (5.0)
1	19 (95.0)
cM, *n* (%)	
0	12 (60.0)
1a	2 (10.0)
1b	3 (15.0)
1	3 (15.0)
Baseline NM staging using conventional imaging	
cN, *n* (%)	
0	3 (15.0)
1	17 (85.0)
cM, *n* (%)	
0	16 (80.0)
1a	1 (5.0)
1b	2 (10.0)
1	1 (5.0)
Preoperative parameters and staging using [^68^Ga]Ga-PSMA-11 PET	
Age (yr), median (IQR)	65.5 (61–69)
PSA (ng/ml), median (IQR)	0.39 (0.04–0.67)
ADT duration (mo), median (IQR)	8.5 (6–12.5)
Docetaxel, *n* (%)	
Yes	11 (55.0)
No	9 (45.0)
Enzalutamide, *n* (%)	
Yes	6 (30.0)
No	14 (70.0)
ycT, *n* (%)	
0	3 (15.0)
2	11 (55.0)
3a	1 (5.0)
3	5 (25.0)
ycN, *n* (%)	
0	13 (65.0)
1	8 (35.0)
ycM, *n* (%)	
0	16 (80.0)
1a	2 (10.0)
1b	2 (10.0)
Pathologic results at surgery	
ypT, *n* (%)	
0	3 (15.0)
2	4 (20.0)
3a	5 (25.0)
3b	8 (40.0)
ypN, *n* (%)	
0	10 (50.0)
1	10 (50.0)
PSM, *n* (%)	
0	11 (55.0)
1	9 (45.0)
LVI, *n* (%)	
0	13 (65.0)
1	7 (35.0)
PNI, *n* (%)	
0	5 (25.0)
1	15 (75.0)
LN removed, median (IQR)	37 (21.5–46)
LN positive, median (IQR)	1 (0–5)
Postoperative PSA (ng/ml), median (IQR)^a^	0.03 (0.01–0.08)

ADT = androgen deprivation therapy; IQR = interquartile range; ISUP = International Society of Urological Pathology Gleason grade group; LN = lymph node; LVI = lymphovascular invasion; PET = positron emission tomography; PNI = perineural invasion; PSA = prostate-specific antigen; PSM = positive surgical margin; PSMA = prostate-specific membrane antigen.

a Measured 6 wk postoperatively.

### 3.1 Primary endpoint

The 2 × 2 contingency tables for baseline and preoperative [^68^Ga]Ga-PSMA-11 PET imaging are shown in Table 2. Sensitivity, specificity, negative predictive value (NPV), positive predictive value (PPV), and diagnostic effectiveness were, respectively, 95.65%, 78.22%, 98.39%, 57.89%, and 83.00% for baseline [^68^Ga]Ga-PSMA-11 PET, compared with 56.52%, 98.05%, 88.30%, 89.66%, and 88.50% for preoperative [^68^Ga]Ga-PSMA-11 PET. Negative and positive LRs were, respectively, 0.06 and 4.39 for baseline imaging, compared with 0.42 and 28.98 for preoperative imaging.

**Table 2:** Contingency table for baseline and preoperative [^68^Ga]Ga-PSMA-11 PET imaging therapy and pathologic results at surgery, after systemic therapy in a region-based analysis of 20 patients

*n* (%)	Imaging neg.	Imaging pos.	Overall
Preoperative [^68^Ga]Ga-PSMA-11 PET imaging			
Histology neg.	151 (98.05)	3 (1.95)	154 (100)
Histology pos.	20 (43.48)	26 (56.52)	46 (100)
Overall	171 (85.50)	29 (14.50)	200 (100)
Sensitivity: 56.52%	Specificity: 98.05%	NPV: 88.30%	PPV: 89.66%
Diagnostic effectiveness: 88.50%			
Baseline [^68^Ga]Ga-PSMA-11 PET imaging			
Histology neg.	122 (79.22)	32 (20.78)	154 (100)
Histology pos.	2 (4.35)	44 (95.65)	46 (100)
Overall	124 (62.00)	76 (38.00)	200 (100)
Sensitivity: 95.65%	Specificity: 78.22%	NPV: 98.39%	PPV: 57.89%
Diagnostic effectiveness: 83.00%			

Neg. = negative; NPV = negative predictive value; PET = positron emission tomography; pos. = positive; PPV = positive predictive value; PSMA = prostate-specific membrane antigen.

**Fig. 1:**
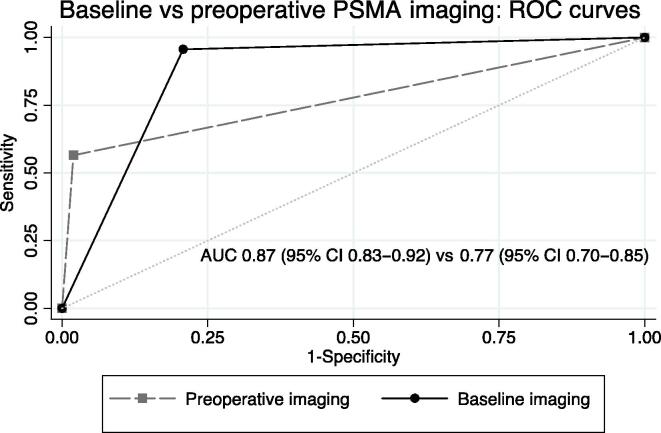
ROC curves of baseline [^68^Ga]Ga-PSMA-11 PET and preoperative [^68^Ga]Ga-PSMA-11 PET after systemic therapy, compared with final histopathology at surgery, in a region-based analysis of 20 patients. AUC = area under the curve; CI = confidence interval; PET = positron emission tomography; PSMA = prostate-specific membrane antigen; ROC = receiver operating characteristics.

On the ROC analysis, the diagnostic accuracy of baseline [^68^Ga]Ga-PSMA-11 PET with an AUC of 0.87 (95% confidence interval [CI] 0.83–0.92) was significantly better than that for preoperative [^68^Ga]Ga-PSMA-11 PET after systemic therapy with an AUC of 0.77 (95% CI 0.70–0.85, *p* = 0.01; Fig. 1).

### 3.2 Secondary endpoints

For morphologic imaging alone, preoperative MRI alone showed similar results to preoperative molecular imaging with sensitivity, specificity, NPV, PPV, and diagnostic effectiveness of 56.52%, 96.75%, 83.87%, 88.17%, and 87.50%, respectively, with an AUC of 0.77 (95% CI 0.69–0.84). There was no statistically significant difference between the diagnostic accuracy of preoperative [^68^Ga]Ga-PSMA-11 PET/MRI and MRI alone on the ROC analysis (*p* = 0.84).

On a per-patient level, including the prostate, baseline [^68^Ga]Ga-PSMA-11 showed sensitivity, specificity, NPV, PPV, and diagnostic effectiveness of, respectively, 100%, 0%, 0%, 85%, and 85%, compared with 82.34%, 66.67%, 40.0%, 93.33%, and 80% for preoperative imaging. When the prostate region was excluded and only pelvic LNs were considered, baseline [^68^Ga]Ga-PSMA-11 showed sensitivity, specificity, NPV, PPV, and diagnostic effectiveness of, respectively, 100%, 20%, 100%, 55.56%, and 60%, compared with 60%, 90%, 69.23%, 85,71%, and 75% for preoperative imaging.

In our exploratory logistic regression analysis, there was a significant association between a pathologic complete response (in the prostate and LNs) and a complete radiographic response on preoperative [^68^Ga]Ga-PSMA-11 PET/MRI (odds ratio 32, 95% CI 1.39–737.56; *p* = 0.03). There was no significant association between pathologic complete response and preoperative PSA, duration of ADT, docetaxel, or enzalutamide. There was also no significant association between pathologic complete response in LNs and PSA, duration of ADT, docetaxel, enzalutamide, or radiographic complete response in LNs (Table 3). As there were no multiple associations, no multivariable analysis was performed.

**Table 3:** Exploratory logistic regression analyses of 20 patients undergoing cytoreductive surgery after systemic therapy with baseline and preoperative [^68^Ga]Ga-PSMA-11 PET imaging

	Logistic regression		
	OR	95% CI	*p* value
PSA			
pCR	<0.001	<0.001–>1000	0.30
pN0	0.60	0.19–1.92	0.39
Duration of ADT			
pCR	1.15	0.94–1.42	0.17
pN0	1.12	0.92–1.36	0.26
Docetaxel			
pCR	0.35	0.03–4.65	0.42
pN0	0.67	0.11–3.92	0.65
Enzalutamide			
pCR	6.5	0.46–91.92	0.17
pN0	2.67	0.36–19.71	0.34
Radiographic complete response			
pCR	32	1.39–737.46	0.03
Radiographic complete response in LN			
pN0	6	0.81–44.35	0.08

ADT = androgen deprivation therapy; 95% CI = 95% confidence interval; LN = lymph node; OR = odds ratio; pCR = pathologic complete response; PET = positron emission tomography; pN0 = pathologic negative lymph nodes; PSA = prostate-specific antigen; PSMA = prostate-specific membrane antigen.

## 4 Discussion

**Fig. 2:**
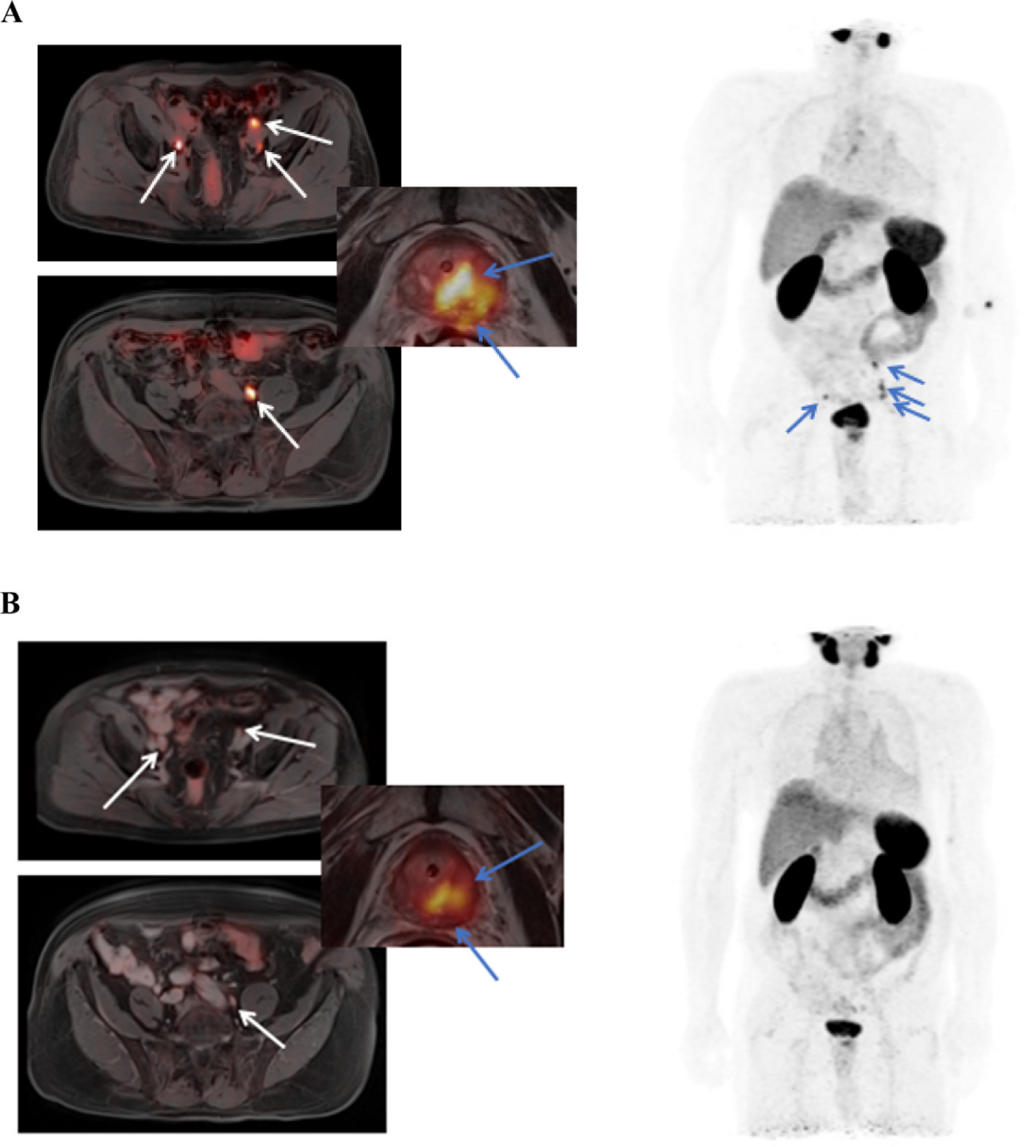
(A and B) Example images of a patient with ISUP 4 disease and PSMA-positive lymph nodes at baseline, which showed a great radiographic response after systemic therapy, yet contained vital tumor at the time of surgery. ISUP = International Society of Urological Pathology; PSMA = prostate-specific membrane antigen.

In this study, we found preoperative [^68^Ga]Ga-PSMA-11 PET/MRI after systemic therapy to have mediocre clinical accuracy on a region-based analysis. However, sensitivity and NPV were not sufficient for basing further treatment decisions, such as the template of resection, on these images. As baseline staging, [^68^Ga]Ga-PSMA-11 PET–based hybrid imaging, however, showed more desirable parameters of diagnostic accuracy with high sensitivity and NPV, and a very low negative LR, for basing further localized treatment on it (example in Fig. 2A and B). On a per-patient level, the results were similar.

Several trials evaluating PSMA-targeted molecular imaging for primary staging have been reported. Even though there is one large prospective evaluation of [^68^Ga]Ga-PSMA-11 PET as baseline staging before surgery reporting very high sensitivity of 85% and an AUC of 0.92 in the proPSMA study [8], most other trials have shown less favorable outcomes. Hope et al. [7] have reported sensitivity and NPV of 40% and 81%, respectively, in a phase 3 trial including 277 patients who underwent RP and extended pelvic lymph node dissection, along with Morris et al. [13] showing sensitivity and NPV of 40.3% and 83.2%, respectively, in their phase II/III trial evaluating [^18^F]F-DCFPyL, another PSMA-targeted tracer. In our study, the PSMA-PET images taken as baseline staging, prior to systemic therapy, not only provided better sensitivity (95.65%), NPV (98.39%), and negative LR (0.06) for the presence of vital tumor tissue in a specific area, than preoperative imaging after systemic therapy, but interestingly were also higher than the values reported for primary staging of patients treated with immediate surgery in any of the previously mentioned studies [2], [7], [8], [13].

One explanation for this could be that additional small tumor foci and micrometastasis, which are missed even by molecular imaging at initial staging, will usually be present on final pathology. Yet these lesions seem to be treated adequately with systemic therapy. This information has the potential to allow for immediate selection of the most suitable candidates for systemic therapy followed by cytoreductive surgery, right at the time of diagnosis. In this case, the extent of resection could be selected at baseline, as all areas with initially positive lesions should be resected; yet, no further extension seems to be necessary.

In the preoperative setting, [^68^Ga]Ga-PSMA-11 PET showed very high specificity for residual disease, and a positive lesion after systemic therapy might be indicative of very aggressive disease; yet, the use of molecular imaging provided no additional information when compared with morphologic imaging using MRI alone. However, while the difference between MRI and CT is considered marginal for the staging of LNs [18], [19], whole-body MRI is also not considered standard and might have reduced the benefit gained from molecular imaging.

Additionally, several studies have established the favorable diagnostic accuracy of PSMA-PET in PCA patients with BCR after local therapy showing superiority over standard imaging in some studies [4], [20], [21], [22]. Abufaraj et al. [11] analyzed PSMA-PET imaging in the setting of BCR in patients undergoing salvage sePLND. The authors also performed a region-based analysis using the same regions as in our study, reporting diagnostic accuracy ranging from 95% to 98% and NPV ranging from 93% to100% depending on the location. Both in primary staging and at the time of BCR, the patients included in these trials had higher PSA levels at the time of imaging than that at preoperative imaging in our cohort. In this regard, in our study, the accuracy of preoperative [^68^Ga]Ga-PSMA-11 PET/MRI before cytoreductive RP was very similar to patients exhibiting similar PSA values in the BCR setting [21] and to patients undergoing primary imaging with immediate surgery.

Currently, it is unclear whether patients with oligometastatic PCA stand to benefit from localized therapy, with the optimal sequence of therapy being currently evaluated in clinical trials. Some ongoing studies include or allow PSMA-based molecular imaging within their protocols. A retrospective analysis of the prospective STAMPEDE (systemic therapy in advancing or metastatic prostate cancer: evaluation of drug efficacy) trial has shown a benefit of local therapy with radiation in patients with low metastatic burden PCA, detected by standard imaging in addition to ADT [23]. Other recently published STAMPEDE data have shown benefit for the combination of intensified systemic therapy with abiraterone, ADT, and local radiation of the prostate, in a very–high-risk localized disease cohort on conventional imaging, which might be similar to a cohort of oligometastatic patients using molecular imaging [24], further supporting the combination of systemic and local therapy for these patients. There are very few reported prospective randomized trials on oligometastatic PCA. Two trials have included patients with recurrent oligometastatic PCA after local treatment and were randomized between observation and stereotactic radiation [25], [26]. In this setting, both Phillips et al. [25] and Ost et al. [26] could show an advantage to metastasis-directed therapy using radiation in terms of progression-free survival. Parikh et al. [27] have initiated a prospective trial evaluating the combination of upfront local treatment with RP, conducted with concomitant ADT for 6 mo and possible radiation therapy. Molecular imaging after 2 mo of ADT is included within their trial. In this and other trials [28], if patients are considered for inclusion and therefore local treatment, complete resection or radiation of all present metastatic lesions is pursued. Surgical resection in the form of sePLND or radiation of LN metastasis, however, comes with substantial morbidity [29]. Ideally, previous systemic therapy and accurate imaging would allow optimization of the extent of local therapy.

To our knowledge, this is the first study describing the diagnostic performance of [^68^Ga]Ga-PSMA-11 PET before and after systemic treatment, with histologic verification. While histology as a reference standard allows valuable insights into this imaging modality, there are many limitations. First, while our database is maintained prospectively, this study was a retrospective analysis. To avoid some of the inherent problems with this design, we used very strict inclusion criteria to evaluate a homogeneous cohort of patients with complete data. However, these criteria and the generally low prevalence of patients presenting with this stage of disease have led to a small number of patients included within this study, which also presents a substantial limitation. There is a selection bias, as patients with immediate disease progression during systemic therapy would not be considered for surgery, thus not be included in our prospective database; anecdotally, this was a very rare event. In addition, patients with bone metastasis were included in our study and histologic verification of these lesions was not feasible. As the intention of this trial was only hypothesis generating, all images and also pathologic specimens were reviewed only by a single specialist in each area; thus, we cannot exclude the presence of interobserver variability.

## 5 Conclusions

As baseline imaging, [^68^Ga]Ga-PSMA-11 PET has significantly better diagnostic accuracy, sensitivity, and NPV than images obtained preoperatively in systemically pretreated patients. If a patient is suitable for local treatment and complete resection of all residual tumor is intended, [^68^Ga]Ga-PSMA-11 PET images taken prior to systemic therapy are significantly more accurate in selecting the relevant LNs for resection.

This imaging modality has the potential to improve our diagnosis as well as guide us for localized therapy at later stages of treatment. Further research on [^68^Ga]Ga-PSMA-11 PET imaging, especially as baseline imaging, in localized high-risk or oligometastatic PCA and its incorporation into prospective trials are needed.

  ***Author contributions:*** Bernhard Grubmüller had full access to all the data in the study and takes responsibility for the integrity of the data and the accuracy of the data analysis.

*Study concept and design:* Huebner, B. Grubmüller, Rasul, Shariat.

*Acquisition of data:* Huebner, Rasul, Baltzer, Clauser.

*Analysis and interpretation of data:* Huebner, B. Grubmüller, Rajwa, Fajkovic, Rasul.

*Drafting of the manuscript:* Huebner, B. Grubmüller, Fajkovic.

*Critical revision of the manuscript for important intellectual content:* K.H. Grubmüller, Mitterhauser, Hacker, Heidenreich.

*Statistical analysis:* Huebner, Baltzer, Rajwa.

*Obtaining funding:* None.

*Administrative, technical, or material support:* Rasul, Clauser.

*Supervision:* Grubmüller, Baltzer, Hacker, Shariat.

*Other:* None.

  ***Financial disclosures:*** Bernhard Grubmüller certifies that all conflicts of interest, including specific financial interests and relationships and affiliations relevant to the subject matter or materials discussed in the manuscript (eg, employment/affiliation, grants or funding, consultancies, honoraria, stock ownership or options, expert testimony, royalties, or patents filed, received, or pending), are the following: None.

  ***Funding/Support and role of the sponsor:*** None.

  ***Ethics statements:*** This retrospective analysis was approved by the Ethics Committee (EC) of the Medical University of Vienna (Ethics Number 1558/2021). All procedures performed in this study were in accordance with the ethical standards of the institutional research committee and with the 1964 Helsinki declaration and its later amendments.

  ***Data sharing:*** The complete dataset used for this analysis is available in a pseudonymized fashion upon request, and after acquisition of IRB approval as well as data sharing agreements between institutions.
