# Supplementary material for: Feasibility and Optimal Time Point of [68Ga]Gallium-labeled Prostate-specific Membrane Antigen Ligand Positron Emission Tomography Imaging in Patients Undergoing Cytoreductive Surgery After Systemic Therapy for Primary Oligometastatic Prostate Cancer: Implications for Patient Selection and Extent of Surgery
Source: Eur Urol Open Sci. 2022 May 5;40:117–24. doi: 10.1016/j.euros.2022.04.003 (PMC9142741; doi:10.1016/j.euros.2022.04.003)
Supplement: Supplementary Data 1 [file mmc1.docx]

## **Supplementary material**

## [68Ga]Ga-PSMA-11 PET/MRI Protocol for patients with prostate cancer

PET/MRI: Biograph mMR (Siemens, Germany) composed of an MRI-compatible PET detector integrated in a 3.0-Tesla whole-body MRI scanner.

Local PET of the pelvis, a 45 minutes dynamic list mode acquisition, started immediately after the intravenous injection of 2 MBq/kg body weight [68Ga]Ga-PSMAHBED-CC conjugate 11 . This followed by whole body PET (skull base to thigh) performed with 4 bed positions, 4 minutes sinogram mode each. Reconstruction parameters for PET were: 3 iterations/ 21 subsets; summation of the 10 minutes pelvic acquisition for visual and semiquantitative analysis. MRI-based attenuation correction was applied using DIXON-VIBE sequences comprising in- and opposed-phase as well as fat- and water-saturated images.

The integrated 3T MRI was performed with the following sequences and parameters: T2 tse tra pelvis: Matrix size 235x512, in-plane resolution: 1.12x0,78x5mm; FoV 262,5x400, TR: 3600ms. TE 106; T2 tse tra p2: Matrix size 320x320, in pl. res. 0.63x0.6x3.5 FoV 200x320mm; TR: 7500ms, TE: 101ms. T2 space tra p2: Matrix 291x320, in-plane resolution 0.7x0.7x1mm; FOV 291x320mm; TR:1600ms, TE: 88ms. T2 tse sag p2: Matrix 310x320, in-plane resolution 0.6x0.6x3.5mm; FoV 200x320mm; TR:7500ms, TE: 101ms. T2 tse cor p2 320: Matrix 320x320, in-plane resolution 0.6x0.6x3.5mm; FoV 200x320mm; TR:7500ms, TE: 101ms. Diffusion weighted imaging: ep2d diff b0 800 tra p2 with Matrix size 132x132, in-plane resolution 1.5x1.5x3.5mm; FoV 200x132mm; TR:4200ms, TE: 87ms. T1 vibe tra dyn dixon 2 means: Matrix 154x192, in-plane resolution 1.4x1.4x3.5mm; FOV 260x192mm; TR:4.75ms, TE: 1.34ms. Whole body MRI simultaneous with PET: T1 vibe fs tra GK KM: Matrix size 195x320, in-plane resolution: 1.2x1,2x3mm; FOV 380x320, TR: 4.56 ms, TE: 2.01. T2w HASTE: Matrix size: 256x256, in-plane resolution: 1.5x1.5x0.6mm; FoV: 380x256mm; TR: 1400ms; TE: 121ms.
